# Finding the Patient’s Voice Using Big Data: Analysis of Users’ Health-Related Concerns in the ChaCha Question-and-Answer Service (2009–2012)

**DOI:** 10.2196/jmir.5033

**Published:** 2016-03-09

**Authors:** Chad Priest, Amelia Knopf, Doyle Groves, Janet S Carpenter, Christopher Furrey, Anand Krishnan, Wendy R Miller, Julie L Otte, Mathew Palakal, Sarah Wiehe, Jeffrey Wilson

**Affiliations:** ^1^ Social Network Health Research Laboratory at the Indiana University School of Nursing School of Medicine, Department of Emergency Medicine Indiana University Indianapolis, IN United States; ^2^ Social Network Health Research Laboratory at the Indiana University School of Nursing School of Nursing Indiana University Indianapolis, IN United States; ^3^ Social Network Health Research Laboratory at the Indiana University School of Nursing School of Informatics and Computing Indiana University Indianapolis, IN United States; ^4^ Social Network Health Research Laboratory at the Indiana University School of Nursing School of Medicine Indiana University Indianapolis, IN United States; ^5^ Social Network Health Research Laboratory at the Indiana University School of Nursing School of Liberal Arts Indiana University-Purdue University at Indianapolis Indianapolis, IN United States

**Keywords:** social meda, health information seeking, adolescent, sexual health, patient engagement, ChaCha, big data, question-and-answer service, infodemiology, infoveillance

## Abstract

**Background:**

The development of effective health care and public health interventions requires a comprehensive understanding of the perceptions, concerns, and stated needs of health care consumers and the public at large. Big datasets from social media and question-and-answer services provide insight into the public’s health concerns and priorities without the financial, temporal, and spatial encumbrances of more traditional community-engagement methods and may prove a useful starting point for public-engagement health research (infodemiology).

**Objective:**

The objective of our study was to describe user characteristics and health-related queries of the ChaCha question-and-answer platform, and discuss how these data may be used to better understand the perceptions, concerns, and stated needs of health care consumers and the public at large.

**Methods:**

We conducted a retrospective automated textual analysis of anonymous user-generated queries submitted to ChaCha between January 2009 and November 2012. A total of 2.004 billion queries were read, of which 3.50% (70,083,796/2,004,243,249) were missing 1 or more data fields, leaving 1.934 billion complete lines of data for these analyses.

**Results:**

Males and females submitted roughly equal numbers of health queries, but content differed by sex. Questions from females predominantly focused on pregnancy, menstruation, and vaginal health. Questions from males predominantly focused on body image, drug use, and sexuality. Adolescents aged 12–19 years submitted more queries than any other age group. Their queries were largely centered on sexual and reproductive health, and pregnancy in particular.

**Conclusions:**

The private nature of the ChaCha service provided a perfect environment for maximum frankness among users, especially among adolescents posing sensitive health questions. Adolescents’ sexual health queries reveal knowledge gaps with serious, lifelong consequences. The nature of questions to the service provides opportunities for rapid understanding of health concerns and may lead to development of more effective tailored interventions.

## Introduction

The development of effective health care and public health interventions requires a comprehensive understanding of the perceptions, concerns, and stated needs of health care consumers and the public at large [1,2]. Clinical and behavioral interventions are most successful when aimed at improving outcomes that are important and relevant to patients. Interventions targeted at these patient-centered outcomes are most effectively developed when patients are engaged in the research process, particularly regarding the identification of salient problems. Funders of health care research increasingly expect proposals to include substantial evidence of attention to patient-centered outcomes through public engagement in the research process, including the process of developing and framing research questions [1-3].

There are many successful models of engaging the public in research, ranging from long-term engagement models such as community-based participatory and action research to the use of focus groups, interviews, and specific designs to elicit stakeholder feedback [4,5]. However, there are substantial challenges associated with these approaches. First, these approaches require a significant investment of time and resources, valued commodities that may not be available to researchers and their teams, nor to communities and their members [6]. Second, in traditional research geographic constraints often limit the number and diversity of individuals who can be included in a single project. Third, most of these methods begin with an a priori research question relevant to the community but often generated by the researcher, which restricts public involvement in the framing of research priorities [7]. In order to overcome the aforementioned limitations and develop relevant and effective patient-centered health interventions, new methods of patient and public engagement are needed.

The Internet has changed the ways in which people seek out and share health-related information [8,9]. Research shows that 35% of Americans report having used the Internet, including social media platforms, to determine what medical condition they or someone they know might have [9,10]. Advances in mobile phone technology make searching the Internet for health-related issues even easier. A recent poll found that 62% of mobile phone owners have used their phone in the past year to look up information about a health condition [11]. Researchers have increasing access to anonymized data from these sites, which have thus far been used to research and disseminate information about disease and disease processes [12]. More recently, social media and other Web-based data sources have been used to facilitate early outbreak detection [13-15]. These datasets can also be used as a point of entry for public involvement in health research. Social media data provide insight into the public’s health concerns and priorities without the financial, temporal, and spatial encumbrances of more traditional community-engagement methods. While these newer methods cannot replace the more traditional ones, social media methods may prove a useful starting point for public engagement in the health research enterprise.

In 2014, the Indiana University Social Network Health Research Laboratory developed a partnership with ChaCha (ChaCha Search, Inc, Carmel, IN, USA) [16], a US-based company that operates a human-guided question-and-answer service that provides free, real-time answers to any question through its website, text messaging, or mobile apps. The data provide a powerful and unique opportunity to listen to the authentic health concerns of individuals. Other Internet-based platforms also provide opportunities to assess population health concerns. Social media platforms have been widely discussed in the literature [17-20]. These platforms, while valuable, are designed for users to communicate with a broad audience of friends or the public at large (eg, Twitter, Facebook), and posts are part of social identity presentation [21]. Conversely, ChaCha queries are a private exchange between an anonymous user and anonymous human guides or a computer. The private nature of the exchange allows users to put forth questions that may be stigmatizing in other settings.

Through our partnership with ChaCha, our laboratory is examining the use of Internet-based question-and-answer services to elicit the patient’s voice and develop health interventions that resonate with public concern. The purpose of this paper is to describe ChaCha user characteristics and health-related queries, and to discuss how this big dataset may be used to better understand the perceptions, concerns, and stated needs of health care consumers and the public at large.

## Methods

In early 2015 we conducted an automated retrospective textual analysis of 1.9 billion anonymous queries submitted to ChaCha by 19.3 million unique users between January 2009 and November 2012. Because we analyzed only existing, de-identified data, the Indiana University Institutional Review Board determined that the study did not meet definitions of human subject research.

We aggregated queries by year in tabulated ASCII text files, in which each line contained 16 data fields representing 1 ChaCha query and 16 associated descriptors (Table 1). Each year’s file was imported to a Linux machine with 64 GB of RAM. Perl scripts were used to parse and summarize the raw data for cleaning and subsequent analyses. A total of 2.004 billion queries were read, of which 3.50% (70,083,796/2,004,243,249) were missing 1 or more data fields, leaving 1.934 billion complete lines of data for these analyses.

**Table 1:** Description of data fields in queries submitted to the ChaCha question-and-answer service.

Field	Description
1	Date and time (eastern time) of query
2	Full category path
3	Auto-detected category
4	Auto-detected subcategory
5	Source type (voice, text message)
6	System used to route and answer question
7	City in which user lives (user reported)
8	State in which user lives (user reported)
9	Region in which user lives (derived from state given in field 8)
10	Country in which user lives
11	Area code of user’s phone number (user reported)
12	Zip code in which user lives (user reported)
13	User’s sex (user reported)
14	User’s age (user reported)
15	User unique identifier (machine generated)
16	Text of query

## Results

### User Characteristics

**Figure 1:**
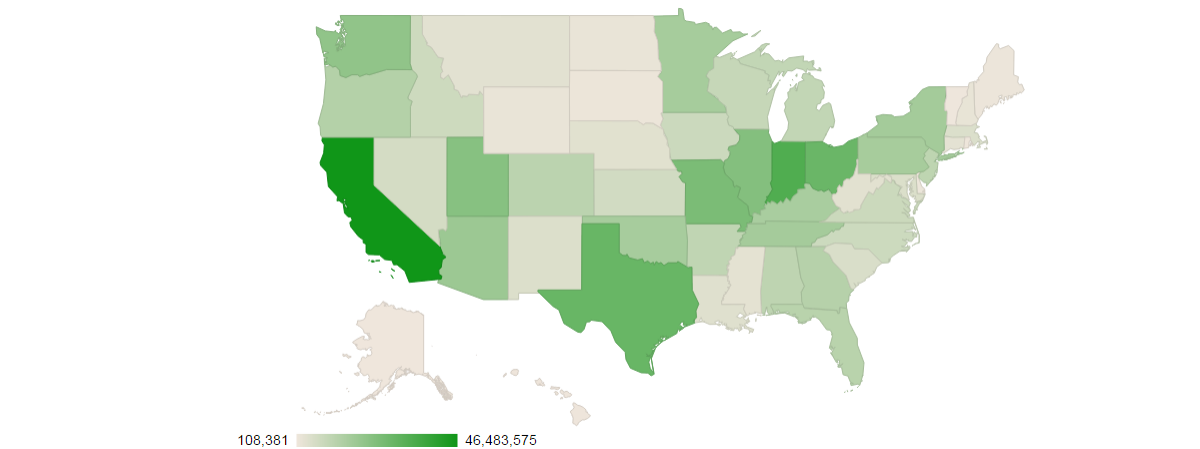
Number of queries posted to ChaCha by user location within the United States, 2009-2012.

There were 19.3 million unique ChaCha users who submitted at least one query during the dates under study. The median user age was 17 years, and approximately 68.35% (5,431,866/7,947,118) of users were younger than age 20 years. There were roughly equal numbers of male (4,367,538/8,875,704, 49.21%) and female (4,508,166/8,875,704, 50.79%) users. The median number of queries per user was 16, with a range of 1–1128 (99th percentile). Approximately 75.93% (1,468,646,207/1,934,159,453) of queries had user profiles from which we could derive the user’s sex, and similarly age from 74.41% (1,439,144,291/1,934,159,453) of queries. A little more than half (800,109,775/1,468,646,207, 54.48%) of these queries were submitted by females. The majority (987,749,753/1,439,144,291, 68.63%) were submitted by users between 12 and 19 years of age. Among these adolescent users, more queries were submitted by females (603,941,883/1,053,718,318, 57.32%) than by males (449,776,435/1,053,718,318, 42.68%). In total, 74.26% (1,436,399,307/1,934,159,453) of queries were made via short message service text message, and the rest from a mix of Web interface, other mobile apps, and voice calls to an automated system. User location (place of residence) was missing for about 73.56% (1,422,701,099/1,934,159,453) of queries. The vast majority of queries were made from the United States (1,933,171,565/1,934,159,453, 99.95%), and approximately 0.05% (987,887/1,934,159,453) of queries originated from the United Kingdom. Figure 1 depicts the user’s location in the United States for the 26.44% (511,458,354/1,934,159,453) of queries for which this information was available.

Service use peaked in 2011, during which there were nearly 672 million queries. Monthly service use fluctuated between 10 million queries in January 2009 and a peak of approximately 60 million queries in May 2011. There were no noteworthy service use trends by month or day of the week. Users most often submitted their questions between 9 PM and 12 AM.

### Content of Queries

All incoming queries were initially filtered a by proprietary ChaCha algorithm that identifies keywords to sort 75.45% (1,459,279,135/1,934,159,453) of queries into 12 broad categories (Table 2) that are further divided into 129 subcategories. Excluding ChaCha customer service-related questions, the queries we analyzed most commonly fell into 5 ChaCha-described categories: (1) Entertainment & Arts, (2) Language & Lookup, (3) Society & Culture, (4) Science & Technology, and (5) Health. Of a total of 106 million health queries, 78.17% (83,056,248/106,254,243) were generated by users who specified their sex and age. We focus here on the subset of those queries (n=68 million) that passed a proprietary ChaCha algorithm that looks for sentence structure, interrogative words, and other factors to filter out “bad questions” that lack sufficient information to be answered.

**Table 2:** Queries submitted to ChaCha: question counts by category and sex (n=1,459,279,135).

Category	Total number of questions	Questions per male user	Questions per female user	% male users, this category	% female users, this category	% of categorized questions (n=1,459,279,135)
Entertainment & Arts	391,911,144	40.2	42.4	49.36% (3,850,766/7,801,869)	50.64% (3,951,103/7,801,869)	26.86% (391,911,144)
Language & Lookup	226,403,804	19.4	25.9	48.49% (3,786,865/7,809,778)	51.51% (4,022,913/7,809,778)	15.51% (226,403,804)
Customer Service	174,889,683	17.5	19.0	49.27% (3,727,948/7,566,817)	50.73% (3,838,869/7,566,817)	11.98% (174,889,683)
Society & Culture	136,908,800	12.5	17.2	48.62% (3,354,359/6,899,650)	51.38% (3,545,291/6,899,650)	9.38% (136,908,800)
Science & Technology	109,703,527	11.1	10.5	49.97% (3,437,238/6,878,206)	50.03% (3,440,968/6,878,206)	7.52% (109,703,527)
Health	106,247,678	11.7	16.4	47.17% (2,847,543/6,036,379)	52.83% (3,188,836/6,036,379)	7.28% (106,247,678)
Sex	89,136,284	15.7	12.6	51.09% (2,587,600/5,064,404)	48.91% (2,476,804/5,064,404)	6.11% (89,136,284)
Lifestyle	74,829,194	8.1	9.5	48.87% (3,095,517/6,334,749)	51.13% (3,239,232/6,334,749)	5.13% (74,829,194)
Politics & Government	47,119,373	6.7	6.6	50.26% (2,436,934/4,848,274)	49.74% (2,411,340/4,848,274)	3.23% (47,119,373)
Sports	46,741,475	9.7	5.1	55.51% (2,617,724/4,715,548)	44.49% (2,097,824/4,715,548)	3.20% (46,741,475)
Business	29,509,62	4.4	4.3	49.60% (2,148,975/4,332,832)	50.40% (2,183,857/4,332,832)	2.02% (29,509,621)
Travel	25,878,552	3.7	4.2	47.93% (2,190,337/4,569,614)	52.07% (2,379,277/4,569,614)	1.77% (25,878,552)

We examined whole-sentence health queries, first those that were generated by roughly equal proportions of males and females, then those that were predominately (≥90%) submitted by females, and finally those predominately (>80%) submitted by males. Among the sex-balanced queries, questions about pregnancy were by far the most prevalent, such as the following: “How are babies made?” “Can you get pregnant on your period?” “What are the signs of pregnancy?” The only other health query frequently submitted by both males and females was about the length of time that alcohol remains in the body.

The queries submitted predominately by females focused on signs and symptoms of reproductive and urinary tract infections, ovulation, and pregnancy. The most common query was about signs and symptoms of yeast infection, followed by inquiries about how to treat, get rid of, or cure a yeast infection. Females more commonly than males asked about the menstrual cycle and its relationship to pregnancy: “When do you ovulate?” “When are you most likely to get pregnant?” “Am I pregnant?” Toxic shock syndrome was frequently mentioned by females, who wanted to know more about its symptoms. Other predominately female user queries included body image questions such as “How can you make your butt bigger?” “How do you get rid of cellulite?”, and 1 relational question: “How do you get over a guy?”

Whole-sentence queries submitted predominately by males focused on body image, particularly penis size and methods for increasing it: “Does ExtenZe work?” “How to make your penis bigger?” “How do I get a six-pack?” Marijuana was the next most-common subject of health queries submitted by males: “What is the best kind of marijuana?” “How many grams in an ounce?” “Why is marijuana illegal?” This was followed by queries related to women’s anatomy and physiology: “How deep is a vagina?” “How do you get a girl pregnant?” Personal health queries focused on testicular discomfort (pain, itching), whether creatine use is safe, and physical fitness goals.

**Figure 2:**
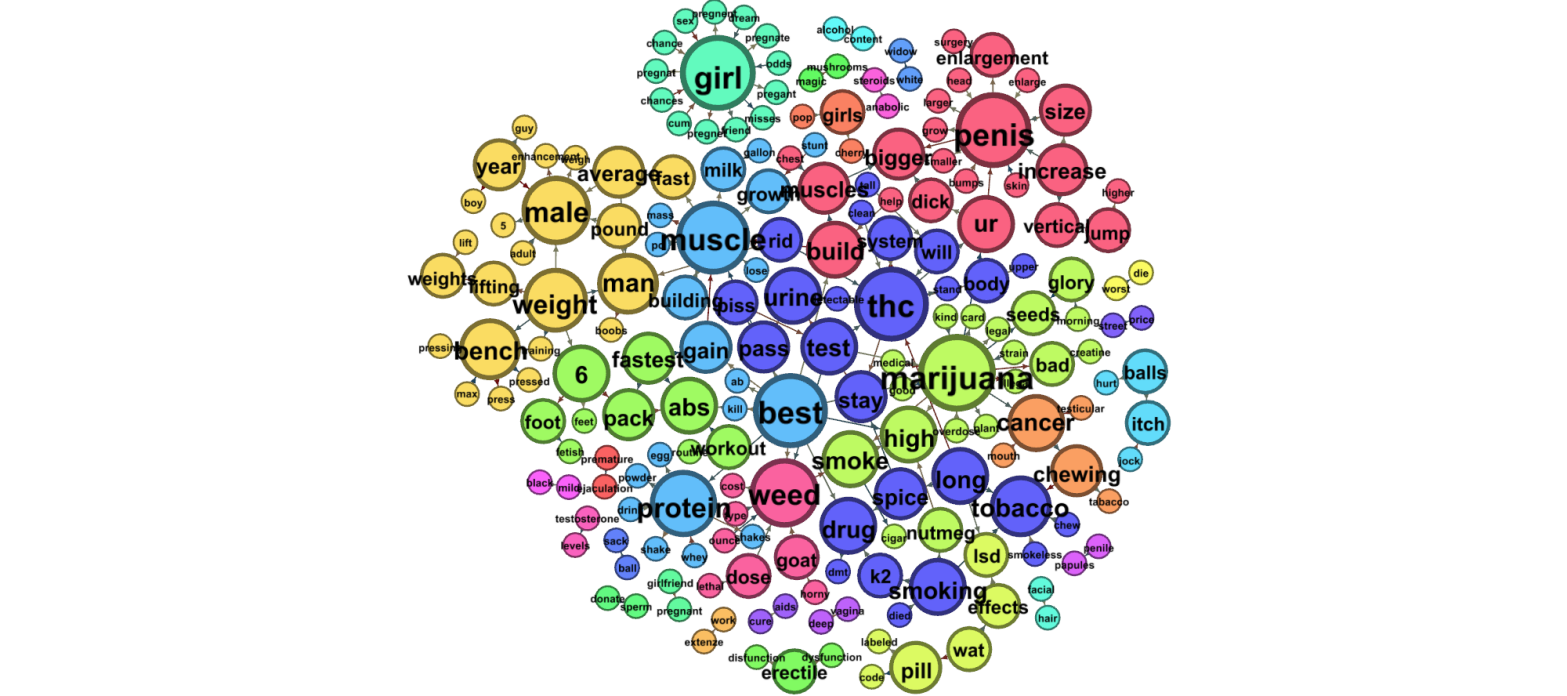
The most prevalent 2-word phrases submitted to ChaCha predominately by male users.

**Figure 3:**
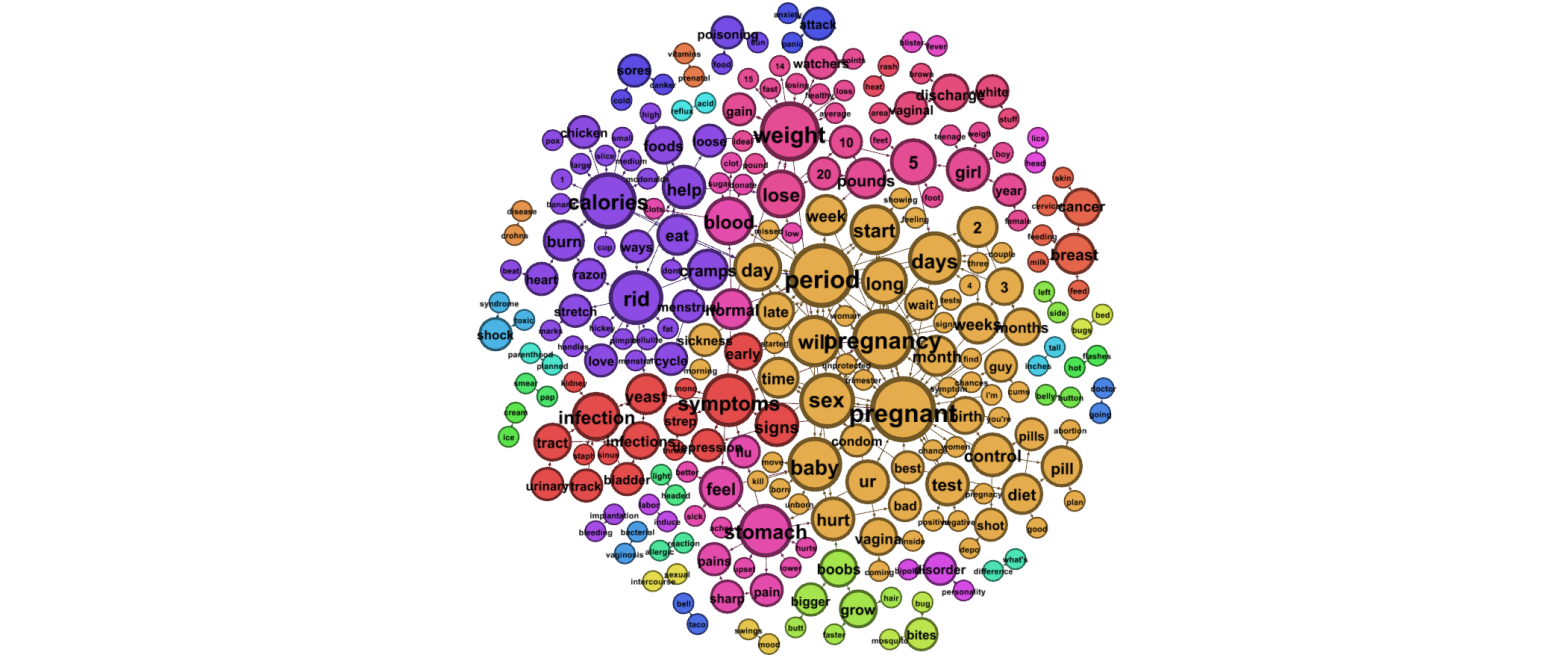
The most prevalent 2-word phrases submitted to ChaCha predominately by female user.

**Figure 4:**
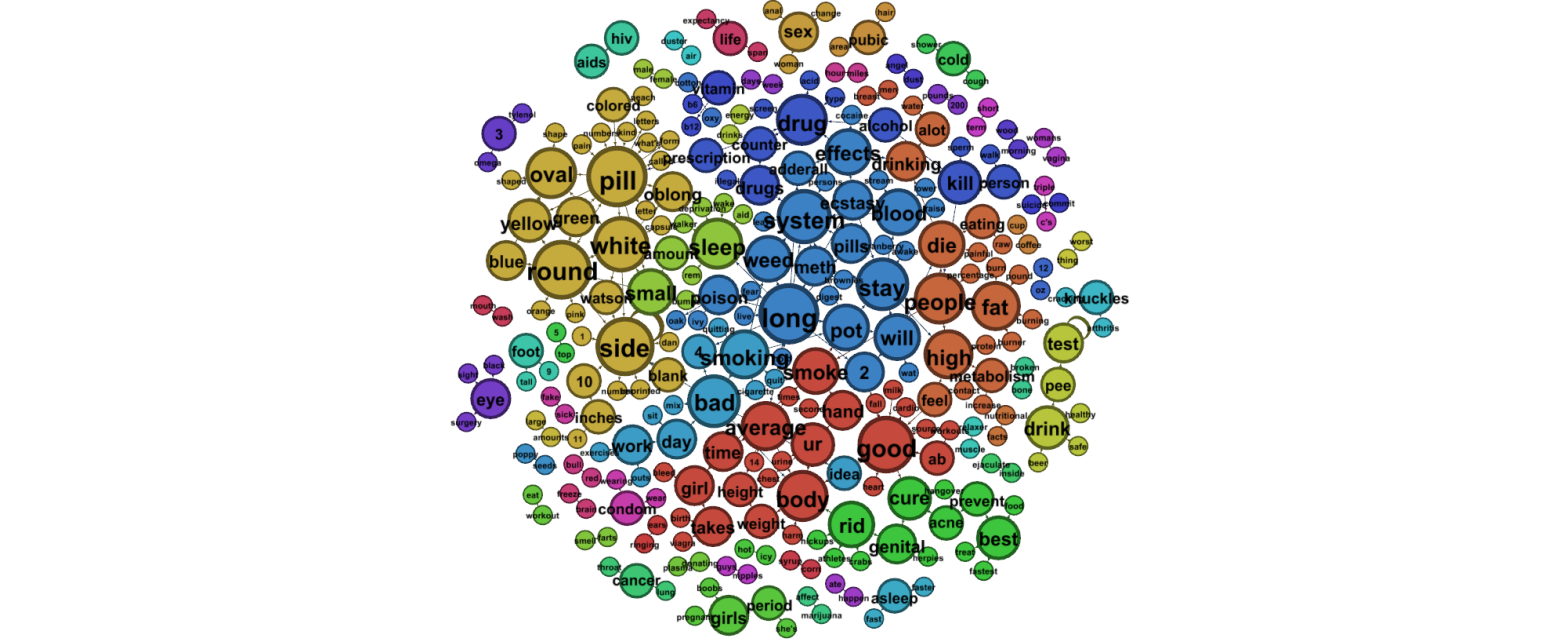
The most prevalent 2-word phrases submitted to ChaCha by both males and females.

Next we examined smaller word groups, of 2- and 3-word phrases, sorted by sex. Table 3 presents the 10 most prevalent 3-word phrases submitted by males, and Table 4 shows those submitted by females. Findings mirrored the whole-word analysis with the addition of weight-loss questions arising in queries submitted by both male and female users. Figures 2 and 3 illustrate the most prevalent 2-word phrases submitted predominately by males and females, respectively. Figure 4 shows the most prevalent 2-word phrases submitted by both males and females.

**Table 3:** The most prevalent 3-word phrases submitted to ChaCha by males.

3-word phrase	Total queries where sex indicated	No. submitted by males	% from males
girl pregnant period	31,670	20,892	65.97% (20,892/31,670)
pass drug test	84,231	55,328	65.69% (55,328/84,231)
stay ur system	24,880	15,944	64.08% (15,944/24,880)
fail drug test	20,372	12,823	62.94% (12,823/20,372)
urine drug test	16,183	10,075	62.25% (10,075/16,183)
kill brain cells	22,120	13,477	60.92% (13,477/22,120)
marijuana stay system	23,891	13,765	57.61% (13,765/23,891)
long marijuana stay	30,325	17,410	57.41% (17,410/30,325)
long-term effects	24,048	13,623	56.65% (13,623/24,048)

**Table 4:** The most prevalent 3-word phrases submitted to ChaCha by females.

3-word phrase	Total queries where sex indicated	No. submitted by females	% from females
symptoms yeast infection	36,158	32,177	88.99% (32,177/36,158)
15 year girl	33,139	28,245	85.23% (28,245/33,139)
early signs pregnancy	49,749	40,368	81.14% (40,368/49,749)
urinary tract infection	91,179	72,242	79.23% (72,242/91,179)
birth control pills	101,851	79,706	78.26% (79,706/101,851)
birth control pill	69,488	53,437	76.90% (53,437/69,488)
help lose weight	79,265	59,683	75.29% (59,683/79,265)
lose weight fast	52,361	39,264	74.99% (39,264/52,361)
pregnant birth control	48,347	33,946	70.21% (33,946/48,347)

**Figure 5:**
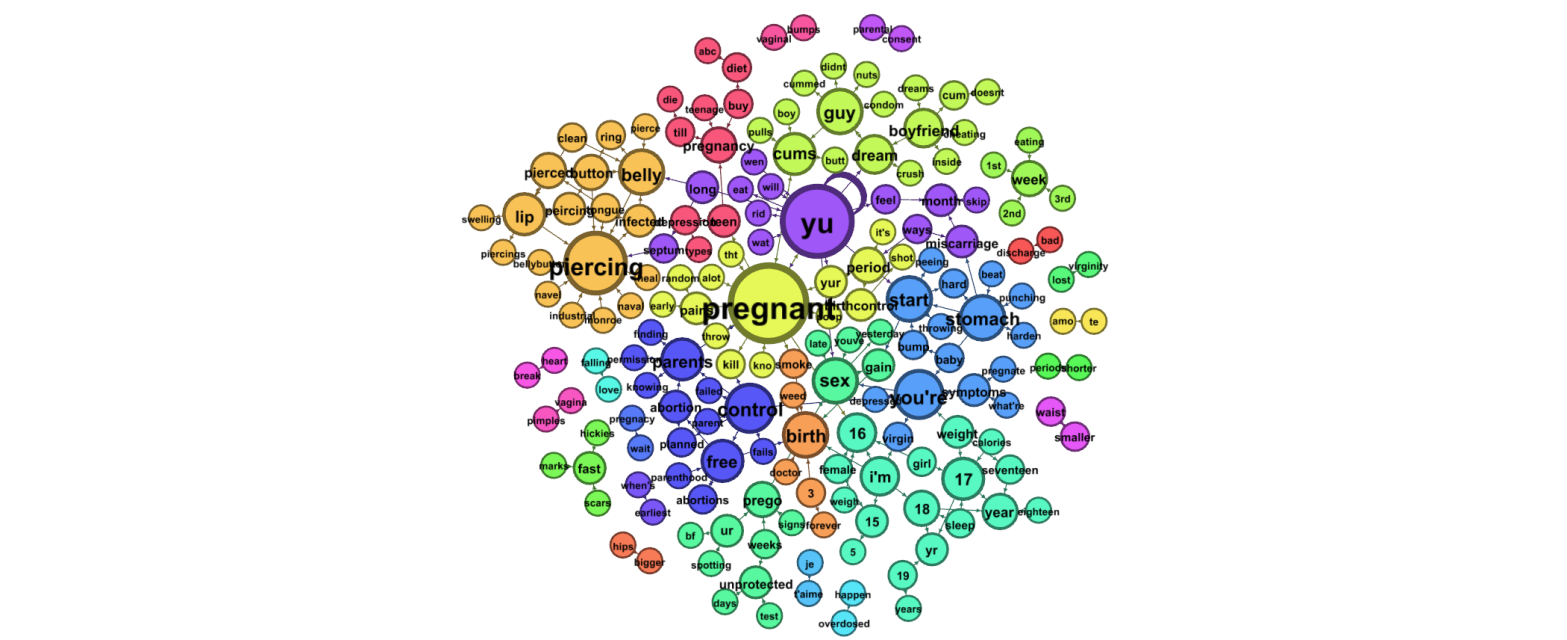
The most prevalent 2-word phrases submitted to ChaCha by users aged 13â€“19 years.

**Figure 6:**
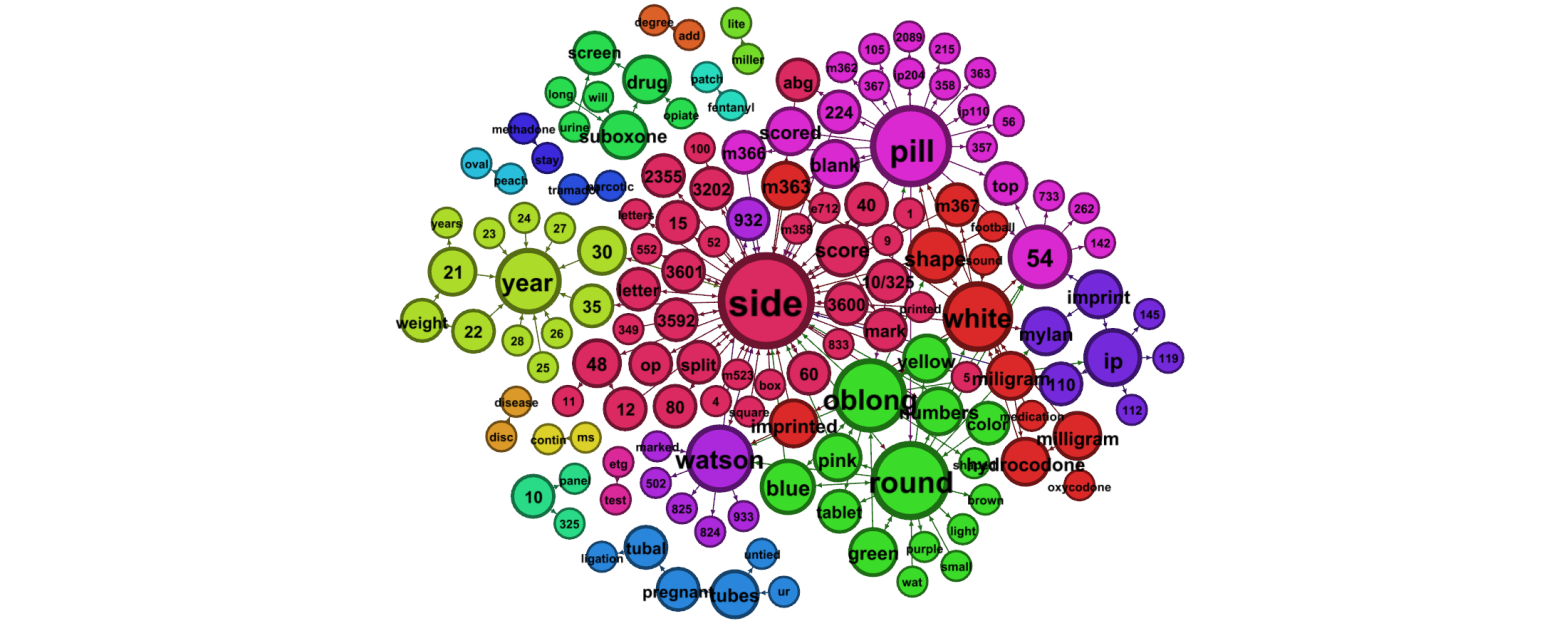
The most prevalent 2-word phrases submitted to ChaCha by users aged 20â€“39 years.

**Figure 7:**
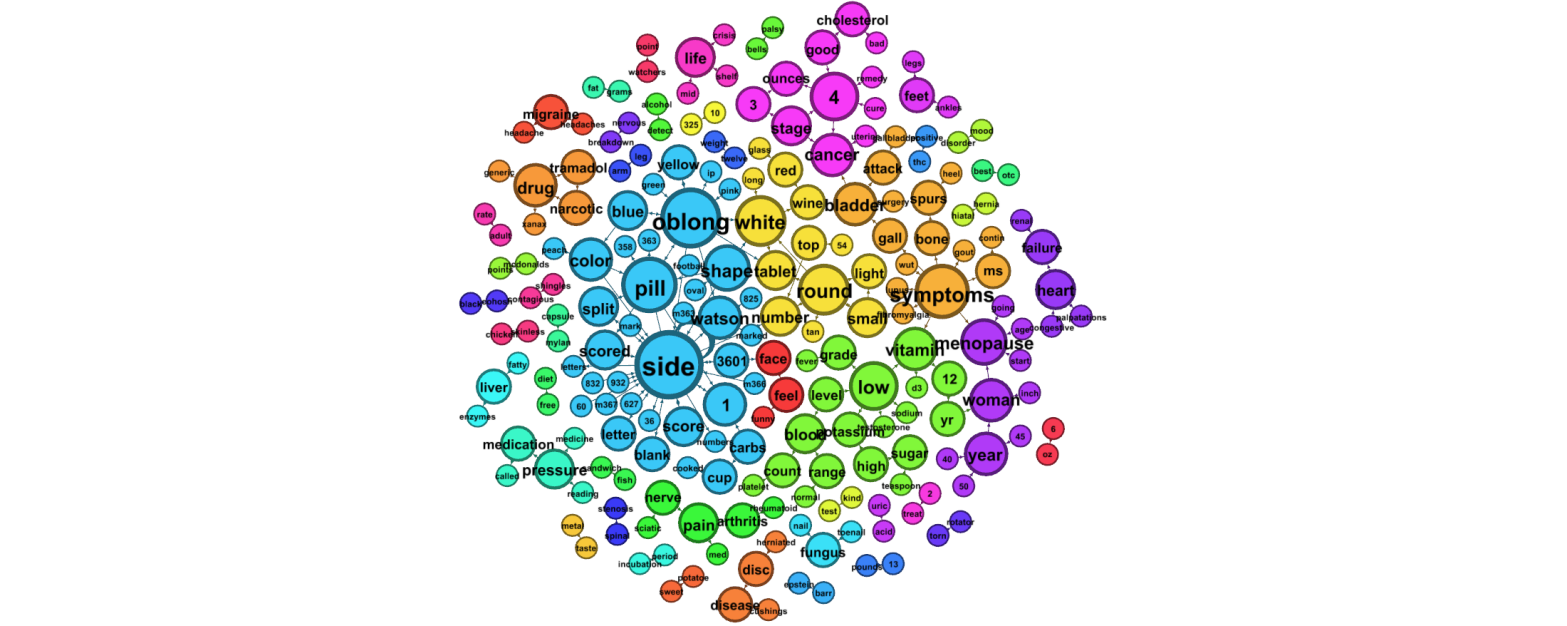
The most prevalent 2-word phrases submitted to ChaCha by users aged â‰¥40 years.

Finally, we examined patterns in queries by age groups. The most prevalent 2-word phrases in queries from users aged 13–19, 20–39, and ≥40 years are depicted in Figures 5-7, respectively.

Among adolescents younger than 19 years, more females than males submitted queries, whereas among young adults aged 19–29 years, more males than females submitted queries. Age patterns were also sex-related patterns, as reflected in the most prevalent 3-word phrases (Table 5).

**Table 5:** Use of 3-word phrases when submitting queries to ChaCha, by sex and age.

Age in years	Males	Females
13–19	average weight 17	17 year girl
	weight 17 year	weight 17 year
	16 year olds	18 year girl
20–39	pill white oblong	pill oblong white
	pill oblong white	pill white oblong
	side blank side	white oblong pill
≥40	white oblong pill	congestive heart failure
	congestive heart failure	side blank side
	13 year girl	small round white

## Discussion

Exploring the ways in which consumers use the Internet to seek health information can also aid Internet-based recruitment for research studies of various types, to improve communication between consumers and health care providers, and to inform the content and geographic scope of marketing for evidenced-based interventions using Internet-accessible platforms. To our knowledge, this is the first analysis of ChaCha data, and these initial results provide valuable methodological and content insights. Methodologically, the results of this initial query affirm our a priori assumption, and the findings of other studies examining Internet health information seeking, that big-data analytical techniques applied to these datasets allow for highly efficient identification of health concerns of users and provide substantial opportunities to develop interventions focused on patient-centered outcomes. Consider that our team analyzed 68 million health-related queries among 1.9 billion overall, generated by 19 million unique users, in less than 5 months and with a total cost of less than $15,000.00. Our entire team working full-time using traditional patient-engagement strategies would have been unable to generate this volume of data in our collective lifetimes, and the cost would be untenable. The ability to analyze such a large volume of user-generated health information-seeking data in such a short time has the potential to fundamentally change patient-centered outcomes research. Patient-engagement strategies are at the heart of effective health outcomes research but are costly and time intensive. Big-data analytic strategies have the potential to make widespread adoption of patient-centered engagement strategies possible at a fraction of the cost.

Several significant content findings from this initial analysis of the ChaCha dataset are consistent with the literature regarding adolescents’ use of social media (eg, Twitter) for seeking health information. The first is that the majority of health-related queries were submitted by adolescent users, which suggests that adolescents are comfortable using an anonymous text-based question-and-answer service for health information seeking, and a similar platform could be useful for interventions targeted to adolescents. The second is that adolescents’ health queries reveal potential knowledge gaps that have serious, lifelong consequences. The vast majority of health questions submitted by adolescents were focused on sexual and reproductive health. They frequently asked about when and how a girl could become pregnant, the signs and symptoms of pregnancy, and the effectiveness and adverse-effect profile of birth control. There were also a large number and proportion of adolescent user-generated queries about the detection and treatment of reproductive tract infections (primarily yeast and urinary tract infections), the length of time that marijuana remains detectable in the blood or urine, weight loss, and wisdom tooth removal. The content of adolescents’ queries indicates their interest in and need for real-time, anonymous answers to questions about their sexual and reproductive health.

As with most studies that analyze social media data, this study had several limitations. First, we do not know whether users were searching for their own knowledge or on behalf of a friend or family member. Second, demographic data were self-reported by anonymous users, who may have misrepresented their city, state, sex, or age. Third, our research team was not provided access to this data until 2014, rendering the data 3–6 years old at the time of analysis. As a result, it is possible that the terminology used to describe health concerns, especially among adolescents, may be slightly outdated. However, we are less focused on *how* people talk about health concerns than on *what issues* cause them enough concern to prompt health information seeking. We believe it is unlikely that the core health concerns raised by users of the ChaCha services have changed dramatically in the last 3–6 years. Importantly, had we applied traditional methods to collect these data, the time lag between collection and analysis would have been substantially longer than the 3- to 6-year gap in our study. Finally, given that this is a proprietary dataset, as are many other social media datasets, it is not convenient for other investigators to replicate this work.

While other question-and-answer services exist, and many are more popular than ChaCha, the ChaCha service has several unique features that make it appealing for patient-centered research. First, ChaCha use is completely anonymous. Users of other question-and-answer sites, such as Quora, are required to sign up for the service using potentially traceable information such as email or Facebook profile. While Quora may be a secure site, the requisite entry of identifiable information in order to use the site may limit the pool of users and the types of questions they are willing to ask. Popular search engines such as Google or Bing provide a greater sense of privacy, but they leave a searchable history, which may also promote self-censorship. Moreover, ChaCha was specifically designed as a question-and-answer service, in which users understood there was a human curating the answers on the other end of the line. This simulates the health care encounter more closely than a Web search, in which the curating is done by the information seeker.

Additional research with these and other social media data are needed to develop a deeper understanding of spatial and temporal patterns in health information seeking that can inform patient-centered research. The ChaCha service provided a perfect environment for maximum frankness, especially around sensitive health questions. Just below the surface of this massive dataset are the quietly whispered questions, both banal and extraordinary, that represent the hopes, fears, dreams, and concerns of millions of people. Without compromising their anonymity in any way, we can listen in, to improve the health and wellbeing of millions more.

## Appendix

Multimedia Appendix 1 The most prevalent 2-word phrases submitted to ChaCha predominately by male users. View interactive graph at .

Multimedia Appendix 2 The most prevalent 2-word phrases submitted to ChaCha predominately by female user. View interactive graph at .

Multimedia Appendix 3 The most prevalent 2-word phrases submitted to ChaCha by both males and females. View interactive graph at .

Multimedia Appendix 4 The most prevalent 2-word phrases submitted to ChaCha by users aged 13â€“19 years. View interactive graph at .

Multimedia Appendix 5 The most prevalent 2-word phrases submitted to ChaCha by users aged 20â€“39 years. View interactive graph at .

Multimedia Appendix 6 The most prevalent 2-word phrases submitted to ChaCha by users aged â‰¥40 years. View interactive graph at .
