# Supplementary material for: Finding the Patient’s Voice Using Big Data: Analysis of Users’ Health-Related Concerns in the ChaCha Question-and-Answer Service (2009–2012)
Source: J Med Internet Res. 2016 Mar 9;18(3):e44. doi: 10.2196/jmir.5033 (PMC4805858; doi:10.2196/jmir.5033)
Supplement: Multimedia Appendix 1 [file jmir_v18i3e44_app1.zip › male/index.html]

OII Network Visualisation Example


 


:   More about this visualisation

## Legend:

## Search:

## Group Selector:

Select Group

Return to the full network

Information Pane

Connections:

OII


JISC
