# Supplementary figures and images for: Finding the Patient’s Voice Using Big Data: Analysis of Users’ Health-Related Concerns in the ChaCha Question-and-Answer Service (2009–2012)
Source: J Med Internet Res. 2016 Mar 9;18(3):e44. doi: 10.2196/jmir.5033 (PMC4805858; doi:10.2196/jmir.5033)

Supplement: Multimedia Appendix 1 [file jmir_v18i3e44_app1.zip › male/images/blank.gif]

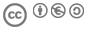

Supplement: Multimedia Appendix 1 [file jmir_v18i3e44_app1.zip › male/images/CC.png]

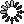

Supplement: Multimedia Appendix 1 [file jmir_v18i3e44_app1.zip › male/images/fancybox_loading.gif]

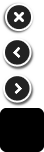

Supplement: Multimedia Appendix 1 [file jmir_v18i3e44_app1.zip › male/images/fancybox_sprite.png]

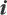

Supplement: Multimedia Appendix 1 [file jmir_v18i3e44_app1.zip › male/images/info.png]

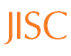

Supplement: Multimedia Appendix 1 [file jmir_v18i3e44_app1.zip › male/images/jisc-logo-small.png]

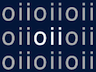

Supplement: Multimedia Appendix 1 [file jmir_v18i3e44_app1.zip › male/images/oii.png]

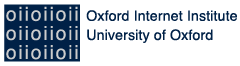

Supplement: Multimedia Appendix 1 [file jmir_v18i3e44_app1.zip › male/images/oii_brand.png]

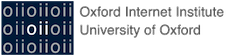

Supplement: Multimedia Appendix 1 [file jmir_v18i3e44_app1.zip › male/images/oii_text.png]

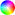

Supplement: Multimedia Appendix 1 [file jmir_v18i3e44_app1.zip › male/images/rainbow.png]

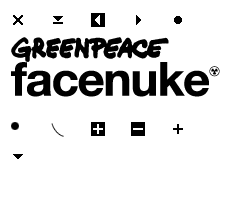

Supplement: Multimedia Appendix 1 [file jmir_v18i3e44_app1.zip › male/images/sprite.png]

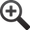

Supplement: Multimedia Appendix 1 [file jmir_v18i3e44_app1.zip › male/images/zoom_in.png]

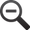

Supplement: Multimedia Appendix 1 [file jmir_v18i3e44_app1.zip › male/images/zoom_out.png]

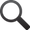

Supplement: Multimedia Appendix 1 [file jmir_v18i3e44_app1.zip › male/images/zoom_reset.png]
